# Neurological examination of Pacific harbor seal (*Phoca vitulina richardii*) pups: development and assessment of a protocol

**DOI:** 10.3389/fvets.2025.1656366

**Published:** 2025-10-01

**Authors:** Christine E. Thomson, Cara L. Field, Sophie T. Whoriskey, Abby M. McClain, Pádraig J. Duignan, J. Margaret Castellini, Marianne Lian, Kris T. Kruse-Elliott, Sophie Dennison, Todd M. O’Hara

**Affiliations:** ^1^Animal Referral Hospital, Brisbane, QLD, Australia; ^2^The Marine Mammal Center, Sausalito, CA, United States; ^3^Department of Veterinary Medicine, University of Alaska Fairbanks, Fairbanks, AK, United States; ^4^Department of Forestry and Wildlife Management, Inland Norway University of Applied Sciences, Elverum, Norway; ^5^AnimalScan and Sage Veterinary Centers, Redwood City, CA, United States; ^6^TeleVet Imaging Solutions, PLLC, Herndon, VA, United States; ^7^Department of Veterinary Integrative Biosciences, College of Veterinary Medicine and Biomedical Sciences, Texas A&M University, College Station, TX, United States

**Keywords:** proprioception, motor function, cranial nerves, reflexes, nociception, neuroanatomical localization, pinniped, phocid

## Abstract

Stranded Pacific harbor seal (HS, *Phoca vitulina richardii*) pups admitted to rehabilitation centers have a variety of health problems, including neurological disorders. However, the standard neurological examination protocol for land-based quadrupeds does not suit marine mammals such as seals. In this study we aimed to develop and establish a neurological examination protocol (NEx) for phocid seal pups undergoing rehabilitation. In two consecutive years, we assessed stranded HS pups (*n* = 45; males *n* = 21, females *n* = 24) in care at The Marine Mammal Center (TMMC), Sausalito, California. The draft protocol developed in year 1 was refined to yield 33 tests, including many tests from domestic small animal examination, as well as novel tests dictated by the animals’ functional anatomy. The latter included the sloping ramp to assess proprioception and motor function, the handstand (to assess neck reflexes), banana pose, and grasp reflex. A fish head was suspended above the subject to assess balance, strength, coordination, and cranial nerve function, including visual field. Specific tests were considered highly useful if they had a reliable outcome in ≥ 80% of cases. In some pups, temperament made it challenging to discern the outcomes of some tests. The reliability of the 33 tests was assessed during repeated examinations on 17 animals; 26/33 tests yielded a reliable response in ≥ 80% of the exams. Three pups (L, P, and N) with suspected neurological deficits were assessed using the protocol. The NEx accurately predicted the neuroanatomical lesion localization, as confirmed by imaging and/or necropsy results. The neurological examination protocol developed for HS pups takes 9–12 min to perform. Although this protocol was developed in HS pups, it should be adaptable for other phocids.

## 1 Introduction

The veterinary neurological examination (NEx) in domestic animals is widely used, effective, and well characterized. It assesses mentation and behavior, gait and posture (comprising proprioception, motor function, and coordination), cranial nerve function, spinal reflexes, nociception, and presence of hyperpathia (1–7). The NEx involves both observation and hands-on testing, and it evaluates the function of both the central nervous system (CNS) and the peripheral nervous system (PNS). The neuroanatomical localization of the lesion is determined by identifying both normal and abnormal functioning of the nervous system. Based on the neuroanatomical localization, signalment, history and physical examination, a list of possible causes (differential diagnoses) is generated, which dictates appropriate diagnostic testing. Assessing the severity of signs and comparing these to known outcomes in similar cases permits a prognosis. Prognosis is particularly pertinent in free ranging animals in rehabilitation where financial and labor costs must be balanced with the likelihood of successful release back to the free ranging population.

The NEx initially developed and applied in pet dogs and cats has been modified for various exotic species, including reptiles (8), ferrets, chinchillas and other rodents (9, 10), and rabbits (11, 12). The examinations are species-dependent and differ according to animal size and age, functional anatomy, behavior and tractability, and available facilities. For example, although “hopping” is a standard part of the examination in dogs and cats, in larger animals such as horses, it is rarely performed. Examination of birds includes specific tests to evaluate their flying ability and the function of the associated highly adapted thoracic limbs (13). In free-ranging animals, observation at a distance is an important part of the assessment because handling may be particularly stressful or dangerous for the animal, the examiner, or both. In domestic animals, performing the NEx relies on the habituation of the subject. Free-ranging animals, when confronted with human interaction, may mask some aspects of neurological dysfunction. Additionally, their behavior and temperament may make responses to stimuli indiscernible.

The aims of the NEx in any species are to determine: (A) if there is a lesion in the nervous system (structural or functional); (B) if so, where the lesion is located (neuroanatomical localization); and (C) whether the lesion is focal, multifocal, or diffuse. The NEx may also indicate the severity of any dysfunction.

An observational protocol to assess neurological function has been developed using stranded Pacific harbor seal (HS, *Phoca vitulina richardii*) pups undergoing rehabilitation before potential release at The Marine Mammal Center (TMMC, Sausalito, CA) (14). This protocol relies on observing pup behavior and mentation (general awareness), locomotion (in and out of the water), interaction with other pups, responses to tactile stimulation, and feeding ability. A lower behavioral score was significantly correlated with eventual time in rehabilitation but did not affect the likelihood of release (14). To enable better characterization of neurological function and hence dysfunction in phocids, we aimed to develop a more detailed, “hands-on” NEx protocol for HS pups. We hypothesized that the standard NEx protocol for domestic species could be adapted for this purpose, but that we may also need to develop novel methods of assessment. The objectives of this study were to determine:

(A) how various functions of the HS pup nervous system could be assessed using testing methods extrapolated from the NEx of domestic animals,
(B) which HS pup neural functions required development of novel testing methods, and
(C) which tests resulted in reliable results. Reliability would be based on ability to discern a positive or negative result in ≥ 80% of animals.

Having a standardized protocol for assessing and recording neurological signs, is key to accurate documentation of how different neurological diseases are manifested. Such data is fundamental for characterizing neurological disorders in animals, such as HS pups, a species in which documenting neurological diseases is difficult. Compared with domestic species, examination of HS pups is more challenging due to them being both free-ranging and marine creatures. We assessed the efficacy of the HS NEx, by applying the protocol to animals with identified clinical, neurological abnormalities.

## 2 Materials and methods

### 2.1 Animals

Free-ranging harbor seal pups under 4 months of age, born free-ranging throughout northern and central California during the regular pupping season of the test year and undergoing rehabilitation at TMMC, were examined over 1-week time periods during the month of May in two consecutive years (*n* = 22, and *n* = 23, respectively). At the time of the NEx, all pups were stable and euhydrated, though in different stages of rehabilitation. For both years, the numbers and presentations of stranded harbor seal pups were similar to historic patterns, with no known harmful algal blooms or other major environmental perturbation overlapping with their admission in either year; specific testing for biotoxins and other common marine toxins was not conducted. All pups were sampled (blood, hair) for mercury levels however results were not received until after the completion of rehabilitation for all seals.

**Figure 1:**
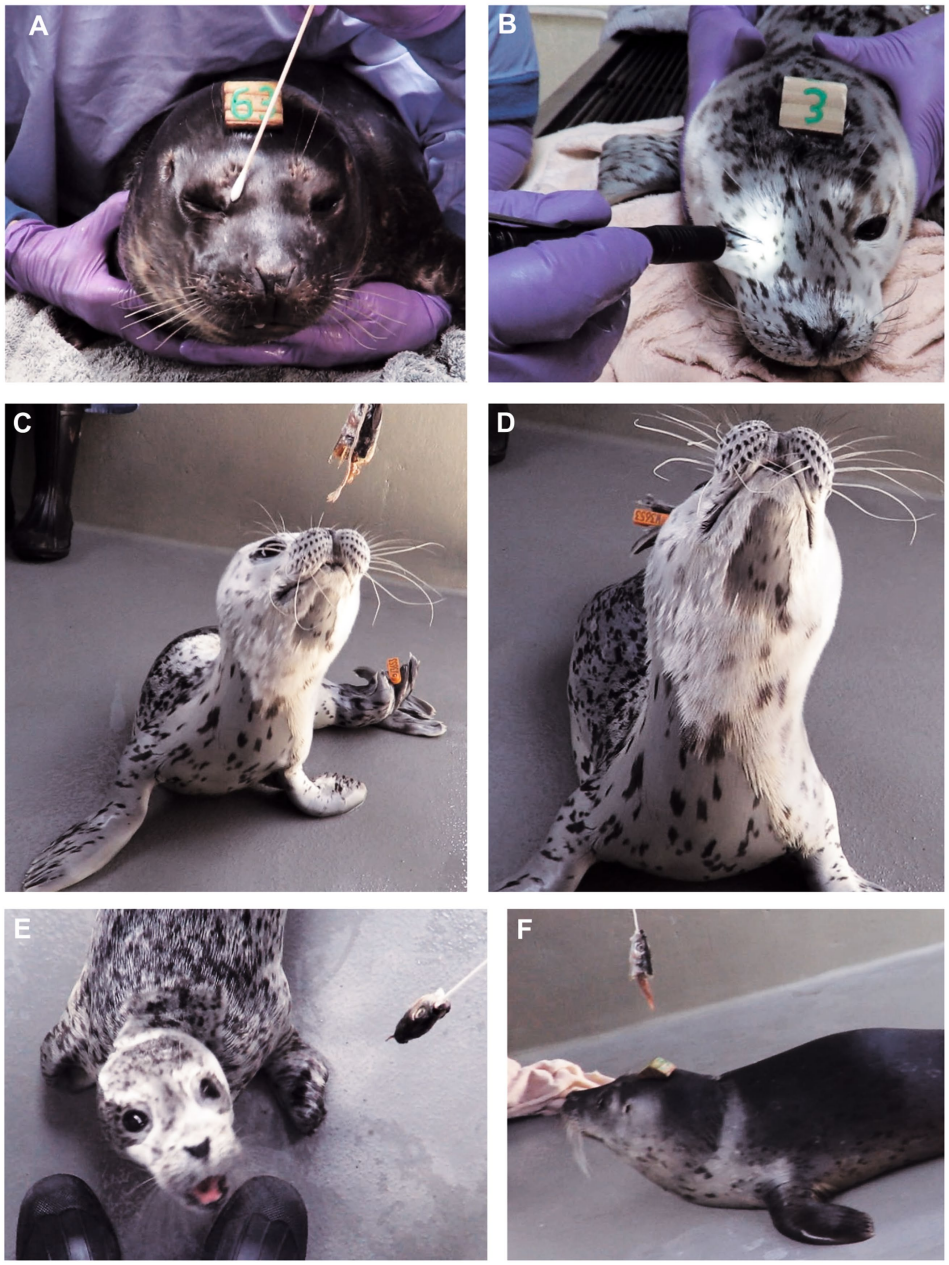
Cranial nerve testing in normal HS pups. **(A)** Palpebral reflex: Stimulation of the medial (or lateral) canthus induced closure of the palpebral fissure. **(B)** Dazzle reflex: Bright light induced closure of the palpebral fissure. The results of both these tests were obvious if the pup was compliant and not vocalizing; when vocalizing they routinely closed their eyelids. **(C)** Extending the head toward a fish head was a good test of visual input and motor output involving balance, coordination and neck strength. **(D)** Pups would actively protrude the mystacial vibrissae toward the fish head (or another stimulus). **(E,F)** Using the fish head to test visual field and vision in normal pups. Starting from behind the animal’s head, the fish head was brought laterally alongside the pup’s head. Normal animals rapidly turned their head to look (exhibited a “visual grasp”) when the object reached a position about 90 degrees lateral to the lateral canthus. Images **(E,F)** are taken from videos showing when the pups first reacted to the visual stimulus; hence the slight blurring.

**Figure 2:**
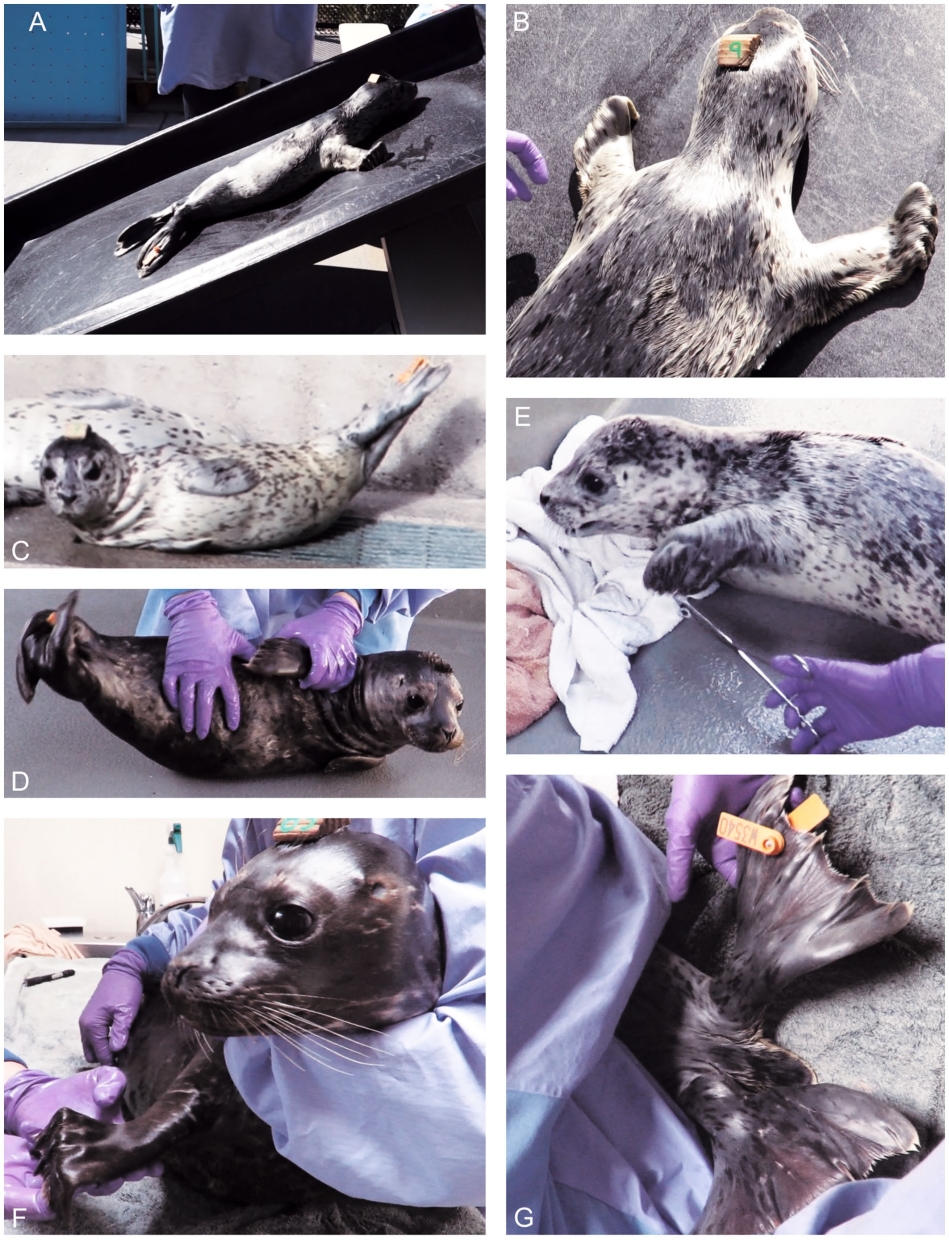
Posture, proprioceptive and motor function testing of normal pups. **(A,B)** Pup on a sloping ramp. Whole-body slipping would induce active flexion of the thoracic limb digits in most pups. **(C)** When resting, pups could adopt a lateral recumbency with scoliosis and elevation of the pelvic limbs. **(D)** When being rolled into dorsal recumbency to assess the righting reflex, pups would actively flex their body laterally. Both the resting and induced scoliosis were termed “banana pose.” **(E)** Gentle squeezing of the flippers (or tail) would induce the flexor withdrawal reflex. **(F)** Manus grasp with digital flexion was induced in most pups by elevating the thoracic limb and stroking the palmar aspect of the manus. **(G)** In 30% of animals, abduction of the pelvic flippers induced active splaying of the digits, especially if the flippers were gently supinated. Tail movement (dorsal or ventral) was noted in 78% of exams. These still images were captured from videos; hence, the occasional blurring.

**Figure 3:**
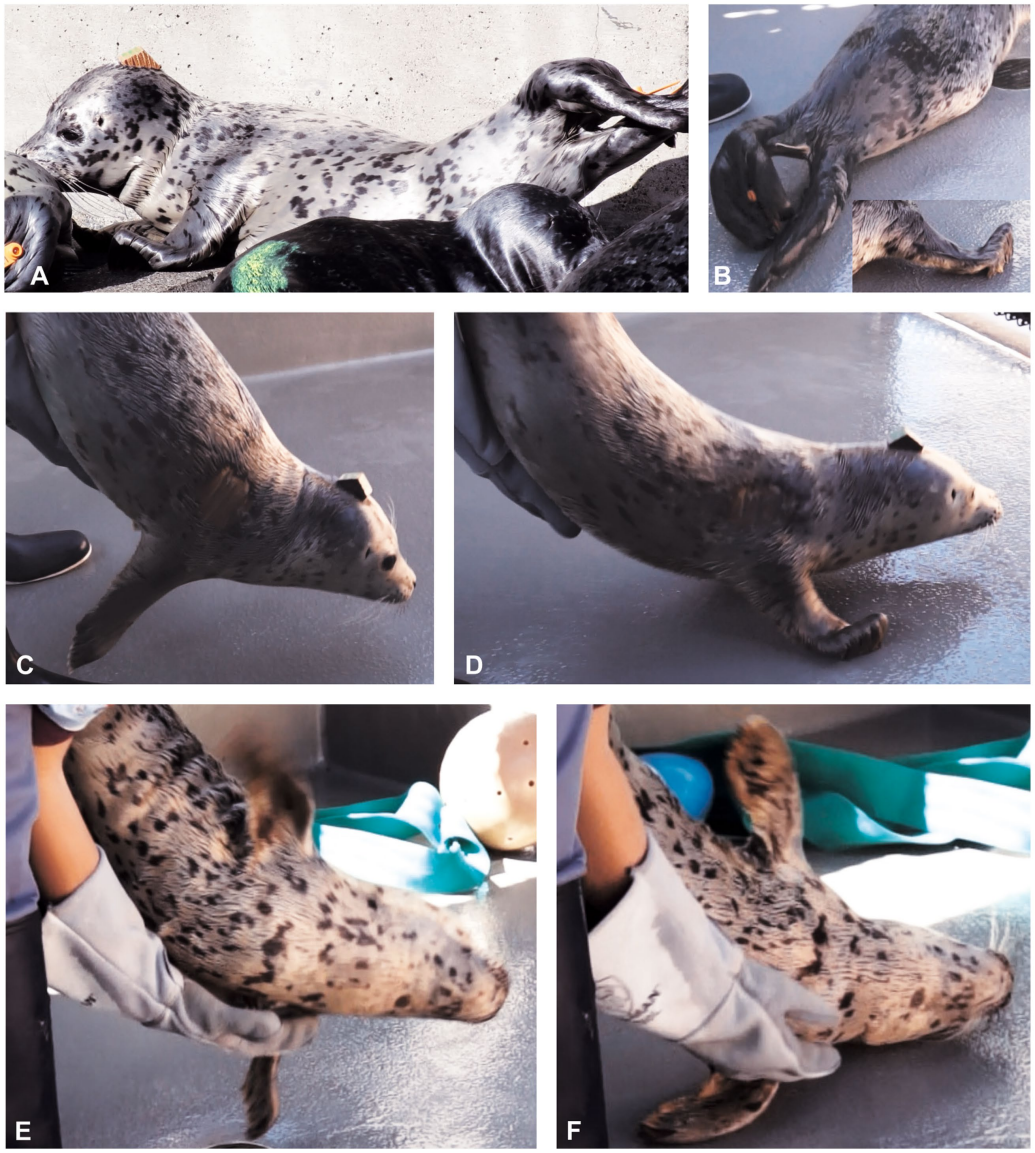
Normal pups lay on their side with some scoliosis and inward curl of their distal pelvic flippers (“banana pose”). **(A)** Pup L appeared spastic and with hypertonicity of the pelvic limb abductor muscles when in “banana pose” (compare with Figure 2C). **(B)** In pup L, elevation of the hind quarters was pronounced during locomotion, as was hyperflexion of the thoracic limb digits (inset in **B**). **(C)** Normal pup tonic neck reflexes (“handstand”) in which lowering the animal toward the ground induced active extension of the neck (so the chin did not contact the ground) and a balanced, stable, suspended posture. **(D)** On contacting the ground, the normal pup would use both thoracic limbs simultaneously, to actively pull itself forward while the hind quarters were still elevated (“wheelbarrowing”). **(E,F)** Animal L had poor balance and coordination. **(E)** He could not sustain the suspended phase of the “handstand” and started tilting left. **(F)** His ataxia induced a “crash landing” on his left side, where he remained for several seconds without righting himself. His righting reflexes were also abnormal. These still images were captured from videos; hence, the occasional blurring.

**Figure 4:**
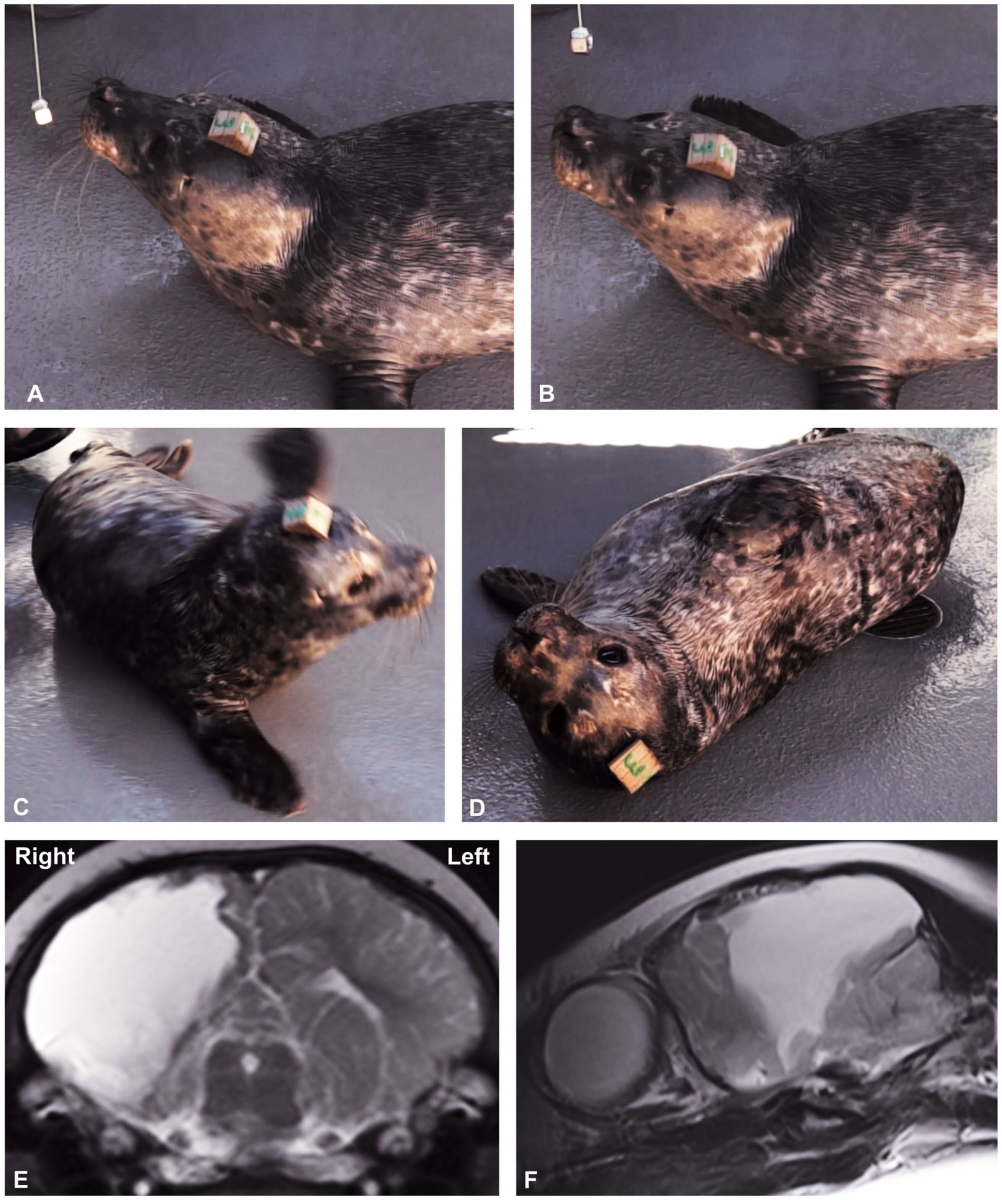
**(A,B)** testing the left visual field in abnormal animal P. **(A)** P has not responded to the object (metal nut) even when it has reached the front of the left eye. **(B)** It was only when the nut was brought further forward over the top of the nose, that it actively looked at it. Presumably this is when it crossed into its right visual field. Still image obtained from video at the point at which she responded to the stimulus. Righting reflex. **(C)** When laid on its back, a normal HS pup can roll well to either side and right itself into sternal recumbency. Animal P’s response to the left was present but inconsistent. **(D)** In Animal P, the reflex was absent rolling to the right, and she remained in dorsolateral recumbency. The still images were captured from videos; hence, the occasional blurring. **(E,F)** Magnetic resonance T2W images of Animal P. **(E)** Transverse slice at the level of the midbrain. (Note: The left side of head is to the right of the image.) **(F)** Parasagittal image of the right cerebral hemisphere. Most of the right cerebral hemisphere is effaced by fluid (white areas) that communicates with the ventricular system, consistent with porencephaly.

**Figure 5:**
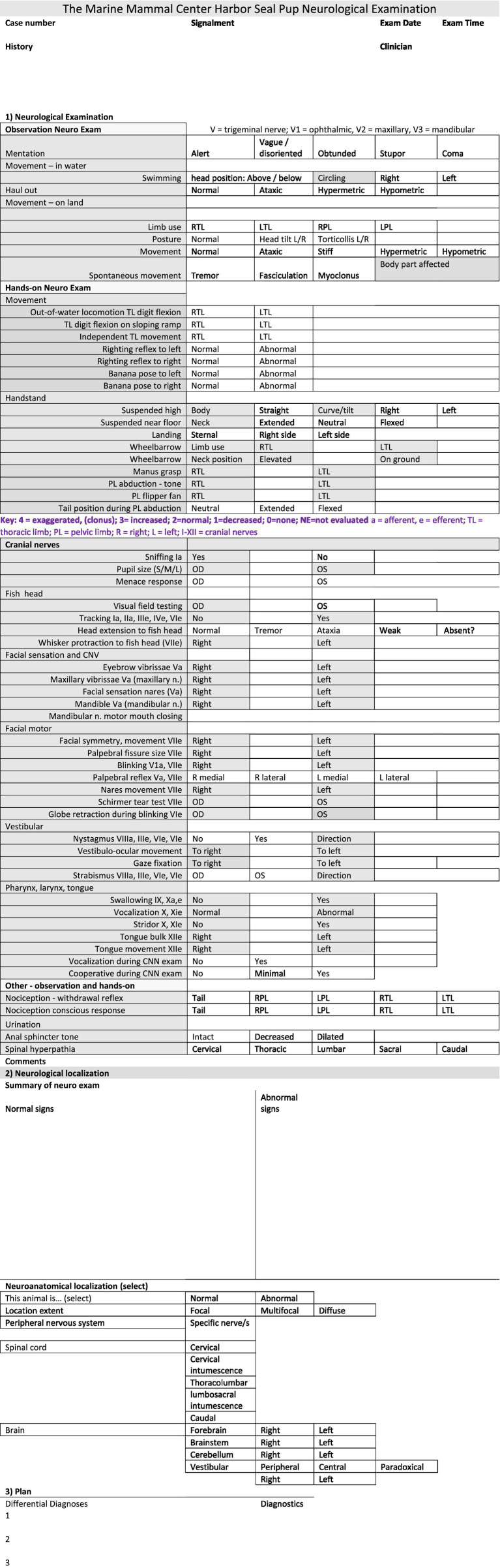
The Neurological Examination Protocol data record sheet. A key at the bottom of the first page lists the abbreviations. After recording the findings (normal and abnormal), it is recommended to summarize them on page 3 and, based on those findings and in conjunction with Table 5, aim to localize the neurological lesion. Lesion localization is the key outcome of any neurological examination. Results of the neurological examination also enable lesion severity to be determined. Based on the neuroanatomical localization, a list of possible causes (differential diagnoses) can be formulated, which directs the diagnostic testing protocol, and, ultimately, the appropriate treatment.

Pup age was estimated by admission date, body mass, pelage, stage of tooth eruption, and presence of an umbilical cord remnant or patent umbilicus (15, 16). All stranded seals were rescued and cared for under National Marine Fisheries Service permit #18786. Standard operating procedures to facilitate internal communications and medical record keeping required that each animal was identified with a unique medical record number, a numbered interdigital hind flipper tag, and a unique name. In this paper, animals are denoted with a specific letter to facilitate reader identification. All animals were housed with between 2 and 7 conspecifics in 6.1 × 4.6 m enclosures with salt-water filled pools (2.4 × 1.8 m, 1.2 m depth with 24–30 ppt NaCl) and were fed a frozen herring diet (*Clupea* spp.) 3–4 times per day. Each animal received a daily intramuscular injection of vitamin B complex during the first 3 days after admission, and daily oral multi-vitamin supplements (Marine Mammal Supplement with Vitamin C, Mazuri, St. Louis, Missouri 63,166, United States) administered during feedings throughout rehabilitation. Upon admission, each animal had a full clinical examination performed by an experienced marine mammal veterinarian and multiple subsequent examinations throughout rehabilitation. Functions assessed during the NEx, included mentation and behavior, cranial nerves (Figure 1), gait and posture, nociception, and spinal reflexes (Figure 2). In some animals, signs of neurological dysfunction were noted (Figures 3, 4). From assessment of both normal and abnormal animals, a neurological examination protocol was established and an appropriate recording sheet developed (Figure 5).

### 2.2 Testing methods

The neurological examinations were recorded on video (Canon, SLX60) for later review by an experienced, board-certified veterinary neurologist. The neurologist (CT) reviewed all videos both at normal speed and in slow motion to improve detection of subtle responses. Videos were used to: (A) establish normal range of responses, (B) analyze consistency between repeated exams in one animal; and (C) establish best testing protocols and refine the test method.

Mentation and behavior were assessed by observing the animal’s response to environmental stimuli, both prior to any stimulation induced by handling and during the hands-on NEx.

Cranial nerve assessment was performed at the beginning of the hands-on NEx, when animals were most likely to be cooperative; however, being free-ranging animals, compliance was quite variable. Each test targets a functional region of the nervous system, and test procedures are presented in Table 1. Visual tracking was noted during observation as the pup moved its eyes either following, or fixating on, a visual stimulus. With the pup gently restrained, menace response, facial sensation and reflexes (e.g., palpebral reflex) were tested using a cotton bud (Figure 1A). A bright penlight was used to assess ocular and visual responses to light, including dazzle reflex (Figure 1B) and pupillary light reflex (PLR). An object dangling from a piece of string was used to assess visual and olfactory responses, CN VII (whisker protraction), neck strength and coordination (Figures 1C–F). Initially, this object was a metal nut but as some pups tried to bite it, this was changed to a fish head. For visual field assessment, the fish head was moved from a position caudal to the animal’s head (behind its visual field) rostrolaterally toward the lateral aspect of the eyes. When it entered the pup’s visual field, the animal would rapidly turn its head to view it.

**Table 1:** Cranial nerve assessment tests used in neurologically assessing harbor seal pups.

Neural function	Region of nervous system utilized	Test(s) and expected response
Sniffing, olfaction	Ia F, BS, cervical SC, cervical SNe	Observation of sniffing, fish head dangled towards the pup
Visual tracking	IIa, F; BS, IIIe, IVe, VIe (extraocular muscles); cervical SC, cervical SNe	Observation of pup tracking a visual stimulus, e.g., person or fish head
Visual field	IIa FB, BS; efferent: BS, cervical SC, cervical SNe	Fish head brought from behind the pup laterally into peripheral vision, pup turns head to observe
Pupil size; pupillary light reflexes (PLR) (direct and indirect)	IIa, F, BS, IIIe	Assess pupil size in bright and dim light, shine bright light in eye assessing pupillary constriction ipsilaterally and contralaterally
Dazzle reflex	IIa, BS, VIIe (narrowing/closure palpebral fissure)	Penlight stimulation of eye, evaluate narrowing of palpebral fissure (squinting)
Menace response	IIa, F, cerebellum, VIIe	Threatening gesture to each eye, normally induces protective response of blinking
Facial sensation Palpebral reflex	Va, VIIe Maxillary n. (lateral aspect) and ophthalmic n. (medial aspect)	Touching lateral and medial ocular canthi, and looking for closure of palpebral fissure
Facial sensation Eyebrow stimulation	Va, probably ophthalmic n.	Touching eyebrow vibrissae, assess eyelid closure
Jaw movement	BS, Ve mandibular n. (VII to caudal digastricus m.)	Observing chewing, biting, opening and closing mouth, vocalization
Facial motor, general facial tone and movement	BS, VIIe	Observing facial symmetry and movement of eyelids, maxillary vibrissae, nares including during respiration and vocalization.
Facial motor, maxillary vibrissae	Voluntary movement maxillary vibrissae: F (presumed motor cortex for voluntary control, to BS and VIIe)	Assessing active protraction of the maxillary whiskers to investigate a stimulus, e.g., fish head being dangled above the pup
Spontaneous blinking	Va (ophthalmic n.), BS,—globe retraction VIe —blinking VIIe	Observing eyelid closure and globe retraction
Vestibular function	VIIIa, BS, Cb, BS, SC, SNe	Observing: Head posture and movement especially when extended (e.g., to fish head and during handstand), head tilt?
Vestibular function	Tactile receptors, SNa, SC, FB, BS, SC, SNe	Righting reflex: turning from dorsal to sternal recumbency. Banana pose: adopting lateral flexion when placed in lateral recumbency
Vestibular function	VIIIa, IIIe, IVe, VIe BS, cervical SC, cervical SNe	Head movement induces vestibular ocular reflex (eye movement). Oculocephalic reflex (gaze fixation then head movement)
Neck motor function and coordination in response to visual stimulus	Ia, IIa, F; BS, Cb, CN XIe, cervical SC, cervical SNe	Dangling fish head, pup reaching to sniff/touch it with maxillary vibrissae. Neck extension during handstand
Pharynx	IXa, Xa; BS, IXe, Xe	Observing swallowing
Larynx	X, XI a; BS X, XI e	Observing breathing, Vocalization
Tongue motor	BS XIIe	May be visible during vocalization

a, afferent (sensory); e, efferent (motor); F, forebrain; BS, brainstem; Cb, cerebellum; SC, spinal cord; SN, spinal nerves. Cranial nerve number in Roman numerals; I, olfactory n.; II, optic n.; III, oculomotor n.; IV, trochlear n.; V, trigeminal n. (maxillary; ophthalmic and mandibular nn.); VI, abducens n.; VII, facial n.; VIII, vestibulocochlear n.; IX, glossopharyngeal n.; X, vagus n.; XI, accessory n.; XII, hypoglossal n. The region of nervous system evaluated (column 2), is based on general mammalian anatomy, using the dog as the type animal. See Methods section for details of the neurological exam tests.

In year 1, assessing the pupillary light reflex was attempted but found to be almost impossible, as the pups’ pupils were observed to be miotic under normal room lighting conditions (normal for this species), and the irises were a deep brown, making it difficult to distinguish the pupillary margin.

Vestibular function was evaluated by assessing whole body posture and balance, with tests such as the: sloping ramp, banana pose, suspended phase of the handstand, righting reflex, neck extension to the fish head, and eyeball position and movement (vestibulo-ocular reflex), in response to head movement (Figures 1–4). However, this reflex was challenging to assess because of both temperament (pups would not permit holding and moving of the head to induce nystagmus), and the pups were actively moving their heads in a variety of directions. It was also difficult to see any globe movement as the white sclera and ocular limbus was hidden beneath the eyelids.

Posture and gait were assessed both by observation and during hands-on testing. This included assessment of proprioception (body position awareness), coordination, muscle tone, and strength of the neck, trunk, limbs, and tail. During the pup’s locomotion out of water, neck position, symmetrical use of the thoracic limbs protracting and retracting to propel the trunk forward, and tone in the pelvic limbs and tail were assessed. Reduced movement (paresis), alterations in muscle tone (hyper- or hypotonia), and ataxia defined as “irregular and mostly unpredictable movement and placement of limbs, head, neck, or trunk” (2) were noted.

Limbs were assessed for posture and movement, both simultaneously and independently. Flexion of the thoracic flipper digits (grip) was noted during locomotion. Initially, we attempted a traditional test of hopping, in which the pup was suspended such that a single thoracic flipper was supporting its weight. The pup was moved craniolaterally to see if the change in proprioception would induce a hopping movement to place the limb into a new weight-bearing position. As doing so proved unreliable, a novel test, named the “sloping ramp test,” was developed using a smooth plastic ramp (1.5 m long by 0.9 m wide) angled at 50–55 degrees (Figures 2A,B). The pup was placed with head towards the top of the ramp so it would gently start sliding backwards due to gravity. It was postulated that slippage should induce stretching of the proximal thoracic limb muscles (extrinsic and intrinsic), especially the shoulder stabilizing muscles. The new muscle and joint proprioceptive input should cause motor responses for gripping, such as contraction of thoracic limb retractor and digital flexor muscles (Figure 2B). Trunk, limb and tail function require appropriate muscle tone and use spinal reflexes. Thus, animals’ locomotion and posture were assessed. The pups were observed resting in lateral recumbency, with hind quarters elevated, the “banana pose” (Figure 2C). This posture could be induced by rolling the animal onto its side on the ground (Figure 2D), which stimulates the vestibular system and reflexively induces increased axial muscle tone on the upper side and lateral scoliosis.

Compared with domestic species the altered pelvic limb anatomy and function in pinnipeds required identification of species-specific techniques for assessing spinal reflexes. Standard patellar (“knee jerk”) and sciatic reflex testing were inappropriate for pinnipeds, however, the pedal/withdrawal reflex in response to a noxious stimulus, was tested as per domestic species, in all four limbs (Figure 2E). The following novel limb function tests were identified and developed. The manus grasp in the thoracic limbs involved holding the pup in sternal recumbency while a second examiner abducted the thoracic limb to a horizontal and slightly cranial position. When the palmar aspect of each thoracic flipper was stroked from carpus to digits, digital flexion (“digital grasp”) was elicited (Figure 2F). With the pup resting in sternal recumbency, the pelvic limbs were gently abducted to assess tone, while simultaneously noting tail position and movement. With the pelvic flippers elevated clear of the ground, they were abducted and pronated. Their plantar aspects were individually stroked, to assess for fanning of the flippers in reflex extension, as in the Babinski reflex (17) (Figure 2G).

Other tests of posture and gait are depicted in Figures 3, 4. Distal flipper posture was assessed at rest and during locomotion. At rest, the thoracic and pelvic flippers in normal pups are relaxed, whereas during movement, distal thoracic flippers (the digits) flex.

The “handstand” involved suspending the pup by its hind quarters and with an examiner’s forearm and hand under the animal’s sternum, tilting the animal 20–40 degrees with head facing towards and approximately 50 cm above the ground. Because of vestibular input from the cranial cervical area and the inner ears, this position should induce neck extension to keep the chin off the ground and stimulate reflex motor function to control whole body posture. The pup was then slowly lowered so it could touch the ground with the thoracic flippers and use them to pull itself forward while the hindquarters were still suspended (“wheelbarrowing”). The handstand and wheelbarrowing tests assess strength and coordination of the cranial half of the body (Figures 3C,D).

The righting reflex was used to assess visual, tactile, and vestibular inputs (18, 19) and motor output to the neck, trunk, and limbs. As the name indicates, this reflex involves the animal righting itself from dorsal recumbency, by rolling over, either left or right, into a ventral or sternal recumbent position. The motor action should be sequential, beginning with head rotation (e.g., to the left) followed by rotation of the contralateral (e.g., right) thoracic limb and trunk and then pelvic limb, from dorsal to ventral (18). The banana pose (Figures 1C,D) and righting reflex (Figure 4C) to both left and right sides were assessed to evaluate the vestibular, coordination and motor function systems.

To assess cutaneous sensation and nociception, initially (year 1) a 10 cm piece of plastic intravenous drip line attached to a pole was used, but in year 2 we switched to using a pair of stainless steel, rounded sponge forceps (Figure 2E). The latter permitted the examiner to better ascertain the stimulus intensity needed to elicit a response and, if necessary, deliver a stronger stimulus to assess nociception. The stimulus was applied dorso-ventrally across each flipper and the tail when the pup was on the ground, unrestrained and not observing the examiner. The intensity of the stimulus applied was the minimum required to induce a limb twitch/withdrawal. We looked for a conscious response such as the pup’s turning to look at the stimulus or moving away from the stimulus.

Post testing, the veterinary neurologist (CT) reviewed batches of all videos captured at the time of the NEx. Test outcomes were assigned to 1 of 3 categories and recorded as follows:

- detectable response (positive)
- no response (negative)
- inconclusive, because it was either not done, difficult to see, or the animal’s temperament and lack of compliance made some responses hard to discern.

We considered that a neurological function test was useful in the HS pup NEx if it yielded a positive result in ≥80% of neurologically normal pups.

### 2.3 Developing the neurological examination protocol

In year 1, standard veterinary neurological function tests were evaluated, and novel tests were developed, leading to a draft protocol that was further tested, modified, and refined the following year with a new cohort of HS pups. In year 2, the order of assessment was: observation, CNN, sloping ramp, fish head, cutaneous sensation, banana pose, righting reflex, and handstand. Some animals were examined once; others were assessed twice, with 1 to 3 days between examinations, aiming to address the following two questions:

(A) Within an individual animal, how many tests yielded a consistent (same) result on two separate occasions? For example, did the pup protract its whiskers to the fish head stimulus on repeated testing?
(B) Across many animals, which tests were yielding consistent results and, therefore, were reliable? For example, was whisker protraction towards the fish head stimulus a consistent response in many animals?

The consistency of the data was stated as a percentage (number of tests with the same results in the pair/total number paired results × 100). Neurological function tests that were reliable (consistent outcome in many animals) were valid for inclusion in the NEx protocol.

### 2.4 Application to animals with neurological deficits—case studies

Six pups were observed by veterinary staff to be abnormal at the time of admission with respect to posture, movement or response to stimuli. These pups were assessed using the NEx. Two pups, L and P, with overt neurological deficits were identified in year 1, when we were still developing the NEx protocol, so testing was incomplete with respect to the final protocol. A third pup, N, was identified in late year 1, while the other three pups (M, T, and G) with possible deficits were identified in year 2. These cases provided important insights as to how confirmed deficits in a phocid may manifest neurologically and could be considered to be opportunistic positive controls (Figures 3, 4). Details of these cases are described elsewhere (20).

## 3 Results

### 3.1 Development and testing of the NEx protocol

In a one-week period in early May year 1, a pilot NEx protocol was developed by evaluating 22 animals. Animal and examination summary is as follows: approximate age range 2–7 weeks; males *n* = 9, females *n* = 13; single exam *n* = 17 animals; repeated exam n = 4 animals; three examinations on *n* = 1 yielding a total number of NEx = 28. Repeat assessments were performed 1 to 3 days apart. A modified NEx protocol was established and tested in year 2.

In a one-week period in early May year 2, the modified NEx protocol was used on 23 animals: males *n* = 13, females *n* = 10; single exam *n* = 6 animals; repeated exam *n* = 17 animals; yielding a total number of NEx = 40. The range of pups’ tenancy at TMMC was 1–7.5 weeks, weight range 8.14-21 kg; and approximate age range 2–7 weeks. Assessments took a total of 9–12 min per animal. Abnormal responses were observed in three animals (section 3.4.2).

### 3.2 Single-exam outcomes

A total of 44/45 (98%) of animals appeared bright, alert and responsive. In year 2, animal G was dull during the NEx, although no health concerns were identified at the time per physical examination, appetite, and lab work, and the animal was ultimately released on reaching an acceptable body weight. Test responses were rated as positive, negative, or inconclusive and are presented in Table 2. The test was considered useful if it yielded a positive result in 80% or more of the pups.

**Table 2:** Results of tests performed during hands-on neurological examinations (NEx).

Gait and posture	Number % positive	Number % no response	Number % inconclusive
Land locomotion PL position	40/100	0/0	0/0
Land locomotion TL digit flexion	34/85	6/15	0/0
TL digit flexion on sloping ramp	34/85	2/5	4/10
*TL hopping*	18/45	6/15	16/40
Neck extension to fish head	38/95	0/0	2/5
Independent TL movement	40/100	0/0	0/0
Righting reflex to left	36/90	0/0	4/10
Righting reflex to right	35/88	0/0	5/12
Banana pose to left	37/92	0/0	3/8
Banana pose to right	36/90	0/0	4/10
Handstand—neck extension	39/97	0/0	1/3
Handstand, wheelbarrow with TL use	38/95	0/0	2/5
Manus grasp	35/88	0/0	5/12
PL abduction—tone	33/83	0/0	7/17
*PL flipper fan during abduction*	12/30	20/50	8/20
Tail movement during PL abduction	25/62	7/18	8/20
Cranial nerves			
Visual field	34/85	0/0	6/15
Visual/olfactory tracking of fish head	39/97	0/0	1/3
*Menace response*	6/15	2/5	32/80
*Dazzle reflex*	21/53	1/3	18/44
Sniffing/tactile to fish head	38/95	0/0	2/5
Whisker protraction to fish head	39/97	0/0	1/3
Nares and maxillary vibrissae movement during respiration	40/100	0/0	0/0
Vocalization during CNN exam	32/80	8/20	0/0
Eyelid, nostril and whisker movement during vocalization	32/80	8/20	0/0
Palpebral reflex	33/83	0/0	7/17
*Maxillary sensation (vibrissae stimulation)*	17/43	3/8	20/49
*Eyebrow sensation (vibrissae stimulation)*	23/58	0/0	17/42
Globe retraction on blinking	40/100	0/0	0/0
Vestibulo-ocular reflex	33/83	0/0	7/17
*Gaze shift*	27/68	0/0	13/32
Nociception			
Cutaneous sensation tail, both PL	39/97	0/0	1/3
Cutaneous sensation both TL	39/97	0/0	1/3

CNN, cranial nerves; ENW, ear, nose and whisker movement; TL, thoracic limb; PL, pelvic limb. Italic font = those tests which yielded insufficient (<80%) positive results. ^1^Gaze shift was only noted as a feature during CNN testing in the last two days year 2 testing, so the positive result may be higher if assessed in more animals.

#### 3.2.1 CNN

Ongoing movement and frequent vocalization by the pups, made performing hands-on NEx of the CNN challenging, with 80% of animals being scored as “non-compliant.” Similarly, blinking during vocalization and head movement made it difficult to consistently assess the menace response, palpebral reflex and dazzle reflex (Figures 1A,B), and response to tactile stimulation of the vibrissae. However, the dangling fish head test was found to be extremely useful as when a fish was held above the pup, 95% would extend their nose to it (Figure 1C), and 98% of those animals actively protracted vibrissae bilaterally (Figure 1D). The visual grasp reflex (turning to look at a new visual stimulus) was elicited in 85% of animals when the fish head was at about 90 degrees to the lateral canthus (Figures 1E,F). Testing the PLR was not pursued.

Vestibular function was evaluated by assessing posture, neck extension to the fish head (Figures 1C,D), banana pose and righting reflex (Figures 2C,D, 4C,D), whole body balance (suspended phase of the handstand, Figures 3C,D), eyeball position, and, where possible, the vestibulo-ocular reflex.

#### 3.2.2 Posture and gait

Posture and gait were assessed by observation during unrestrained movement, and challenged during the hands-on NEx by putting the pup in novel positions: on the sloping ramp, banana pose, righting reflex, handstand (Figures 2A–C, 4D; Table 2) and using the dangling fish head (Figures 1C,D). Eighty-five percent of pups actively flexed the digits of the thoracic flipper (digital grasp) either during locomotion, on the sloping ramp, or both (Figures 2A,B; Table 2). The manus grasp was inducible in 88% of animals by passively abducting the thoracic flipper and stroking the palmar aspect of the manus (Figure 2F; Table 2). Thoracic flipper digital flexion was not always present on all three assessments (sloping ramp, during locomotion, stroking of the flipper); however, in 39/40 (98%) of examinations in year 2, it was present in at least one test, demonstrating the utility of employing this suite of tests rather than a single test. Pelvic limb tone and movement were assessed by abducting the pelvic flippers (Figure 2G), which induced tail movement in 25/32 (78%) exams in which it was recorded (6 dorsal movement, 19 ventral movement). Stroking the plantar aspect of the pelvic flipper induced digital extension (fanning of the flipper) in only 30% of animals (Figure 2G). Balance, coordination, and strength of the cranial half of the body, involving vestibular, cerebellar, and motor function, were readily assessed. Neck and thoracic limb proprioceptive and motor function were assessed during spontaneous locomotion, dangling fish head (Figures 1C,D), sloping ramp (Figures 2A,B), and the suspended and wheelbarrow phases of the “handstand” (Figures 3C,D).

#### 3.2.3 Spinal reflexes and nociception

The main spinal reflex assessed was the withdrawal reflex, which was stimulated by applying a potentially noxious stimulus dorso-ventrally across each of the four flippers and the tail. The stimulus intensity used was sufficient to produce only a partial withdrawal of the limb (Figure 2B, 100% positive, Table 2). In one animal, M (see section 3.4.2), a stronger, more sustained stimulus was required.

### 3.3 Repeated exam outcomes

Repeated examinations enabled assessment of (A) the consistency of a test result within an individual animal and (B) the reliability of a specific test to yield consistent results across many animals.

Whether a neurological function test yielded consistent results was determined by applying that test twice in the same animal (Table 3). If the outcome of testing was the same, test results were consistent. Between 29 and 33 of the neurological functions identified in Table 2 were tested twice in 17 animals. For each animal, the number of neurological functions with consistent results over the total number of functions tested was determined, yielding a percentage value. Of the 17 animals, 14 had more than 82% of tests yielding consistent results, two were 81% and one was 77% (animals J, T, and M, respectively). These lower scores could indicate clinical deficits (Animal M and T) or could be attributed to a lack of cooperation rather than decreased or abnormal responses (Animal J) (see section 3.4.2).

**Table 3:** Consistency of test outcomes within individual animals.

Neurological test	Consistency/number of animals with paired assessments	Consistency of assessments %
Gait and posture		
Land locomotion PL position	17/17	100
Land locomotion TL digit flexion	12/17	71
TL digit flexion on sloping ramp	14/15	93
Neck extension to fish head	15/17	88
Independent TL movement observed	17/17	100
Righting reflex to left	14/15	92
Righting reflex to right	11/13	85
Banana pose to left	14/15	93
Banana pose to right	15/15	100
Handstand—neck extension	15/16	94
Handstand, wheelbarrow with TL use	14/15	93
Manus grasp	12/13	92
PL abduction—tone	11/12	92
*PL flipper fan during abduction*	6/12	50
*Tail movement during PL abduction*	5/12	42
Cranial nerves		
Visual field	13/13	100
Visual/olfactory tracking of fish head	16/16	100
Dazzle reflex	10/10	100
Sniffing/tactile with whiskers to fish head	16/16	100
Whisker protraction to fish head	16/16	100
Nares and maxillary vibrissae movement during respiration	17/17	100
Compliance during cranial nerve exam	15/16	94
Eyelid, nostril and whisker movement during vocalization	12/12	100
Palpebral reflex	12/12	100
Maxillary sensation (vibrissae stimulation)	2/2	100
Eyebrow vibrissae stimulation	8/8	100
Globe retraction on blinking	17/17	100
Vestibulo-ocular reflex	10/10	100
*Gaze shift*	8/13	62
Nociception		
Cutaneous sensation tail, both PL	15/17	88
cutaneous sensation both TL	15/17	88

In year 2, repeated neurological examinations (NEx) were performed on 17 animals, 1–3 days apart, using 29–33 of the tests of neural function (as specified in Table 2). Some tests could not be performed or yielded inconclusive results because of individual animal temperament and/or lack of compliance. For the tests that could be performed, the consistency of those tests over the total number of tests performed, are denoted both as total values and percentages.

Whether individual tests were reliable was determined by assessing which individual tests gave consistent outcomes across many animals (*n* = 17) (Table 4). Individual tests that were identified as unreliable (having low consistency on paired examinations in many animals) included whether: (1) the animal flexed its thoracic flipper digits during out-of-water locomotion (12/17 = 71%), (2) the pup vocalized during the cranial nerve examination (12/16 = 75%), (3) the pelvic flippers fanned out when stroked (6/12 = 50%), and (4) tail movement was induced by pelvic limb abduction (5/12 = 42%). Thoracic flipper hopping and menace response were not assessed due to low positive responses (18/45 = 40% and 6/15 = 40%, respectively) identified during first phase of study in year 1.

**Table 4:** Reliability of specific neurological tests across many animals.

Neurological test	Consistency/Number of animals with Paired Assessments	Consistency of Assessments %
Gait and posture		
Land locomotion PL position	17/17	100
Land locomotion TL digit flexion	12/17	71
TL digit flexion on sloping ramp	14/15	93
Neck extension to fish head	15/17	88
Independent TL movement observed	17/17	100
Righting reflex to left	14/15	92
Righting reflex to right	11/13	85
Banana pose to left	14/15	93
Banana pose to right	15/15	100
Handstand—neck extension	15/16	94
Handstand, wheelbarrow with TL use	14/15	93
Manus grasp	12/13	92
PL abduction—tone	11/12	92
*PL flipper fan during abduction*	6/12	50
*Tail movement during PL abduction*	5/12	42
Cranial nerves		
Visual field	13/13	100
Visual/olfactory tracking of fish head	16/16	100
Dazzle reflex	10/10	100
Sniffing/tactile with whiskers to fish head	16/16	100
Whisker protraction to fish head	16/16	100
Nares and maxillary vibrissae movement during respiration	17/17	100
Compliance during cranial nerve exam	15/16	94
Eyelid, nostril and whisker movement during vocalization	12/12	100
Palpebral reflex	12/12	100
Maxillary sensation (vibrissae stimulation)	2/2	100
Eyebrow vibrissae stimulation	8/8	100
Globe retraction on blinking	17/17	100
Vestibulo-ocular reflex	10/10	100
*Gaze shift*	8/13	62
Nociception		
Cutaneous sensation tail, both PL	15/17	88
cutaneous sensation both TL	15/17	88

TL, thoracic limb; PL, pelvic limb. In year 2, specific neural function tests were assessed for consistency in up to 17 animals. The tests were considered consistent at ≥ 80%. Because of subject noncompliance, some tests (e.g., palpebral reflex, maxillary and eyebrow vibrissae sensation) were successfully performed in only a few animals. Italics indicate reliability of <80%.

Those tests with ≥80% reliability were considered valid for inclusion in the neurological testing protocol and hence were included in the data recording sheet for the NEx for HS pups (Figure 5). Note, tests 1–4 above, vestibulo-ocular reflex and menace response, were still included on the NEx protocol, despite lower reliability, as observing a confirmed result may still be useful.

### 3.4 Case studies assessing efficacy of the NEx for determining the lesion localization

#### 3.4.1 Year 1

##### 3.4.1.1 Case summaries

Animal L, a 2–3 week old pup at time of stranding, displayed ataxia, incoordination, perturbed balance, spasticity, and tremors. Spasticity was indicated by sleeping in hyper-flexed lateral recumbency with the proximal pelvic limbs abducted and flippers curled inward (Figures 3A,B). This animal’s movements were ataxic and clumsy, with persistent hyperflexion of the thoracic flipper digits (Figure 3B inset). The righting reflex was repeatedly incomplete to the right, and the pup remained lying in left lateral recumbency; the righting reflex to the left was normal. Compared with those of a normal pup (Figures 3C,D), the handstand and wheelbarrow responses were abnormal. During the suspension phase of the handstand, it was ataxic and wobbling from side to side. As it was lowered to the floor, the pup would tilt left and land clumsily on the left side, where it remained with incomplete righting back to the normal sternal posture (Figures 3E,F). These signs were consistent with dysfunction of the cerebellar and vestibular systems. Due to the persistently abnormal clinical presentation, an MRI was performed after 24 days in care and was interpreted by a board-certified veterinary radiologist with extensive experience in marine mammal diagnostic imaging (SD). The MRI demonstrated fluid accumulation within the tympanic bullae consistent with otitis media, marked medial retropharyngeal and mandibular lymph node enlargement (most suggestive of reactive lymphadenopathy), and intramedullary pathology of the spinal cord at the level of C1-2, with differentials of myelitis, developing syringomyelia, or gliosis. Balance and vestibular function may have been compromised by both the middle ear fluid accumulation and the C1-2 lesion, with the latter potentially affecting spinocerebellar and spinovestibular tract function. The pup responded well to antimicrobial treatment, as evidenced by clinical resolution on subsequent clinical assessment and resolution of lesions on repeat MRI. It was eventually released after a total of 10 weeks in care.

Animal P, a < 7 day old pup, was observed to have behavioral changes including erratic and repetitive swimming, often with its head out of the water, and rapid shifts in mentation from subdued to aggressive. On NEx, it was unresponsive to stimulation in the left visual field, suggesting blindness in the left eye (Figures 4A,B). The pup did not flex the thoracic flipper digits on the sloping ramp, despite sliding all the way down it. Upon light cutaneous stimulation, P had less response from the left pelvic flipper than the right. However, it did have symmetrical response to truncal and facial tactile stimulation (eyebrows and mystacial vibrissae). The pup had respiratory and voluntary movements of the mystacial vibrissae (bilaterally), but between movements, the left mystacial vibrissae were held more retracted than on the right side. The righting reflex was inconsistent to the left and absent to the right (Figures 4C,D), and it remained lying in dorsolateral recumbency. While P was lying upside down, several beats of pathological nystagmus were observed. Key clinical signs noted on the NEx, included deficits in the left visual field, left tactile sensory deficits, and impaired voluntary left facial movements. Along with the mentation change and altered voluntary movement, these signs were consistent with a neuroanatomical localization of the right forebrain, potentially including the vestibular projection to the right temporal lobe. Alternatively, the pup could have had a multifocal lesion affecting the visual and vestibular systems.

Magnetic resonance imaging was performed after 31 days in care and interpreted by SD. Porencephaly (fluid-filled cavities in the cerebral hemispheres) was identified affecting the right temporal, parietal and occipital lobes. (Figures 4E,F). Given the extensive abnormalities combined with clinical decline over time, this animal was humanely euthanized. Pathological changes identified on MRI were confirmed at necropsy. On histopathology, the right cerebral peduncle and pyramid (efferent tracts from the right cerebrum) were reduced in size. The clinical blindness in the left eye was consistent with the lack of visual cortex on the right side.

Animal, N, a 3–3.5-month-old pup, was assessed using some aspects of the draft NEx protocol in November year 1. Animal N was severely underweight and had suspected bilateral blindness. The pup had no response to visual field testing, and neither tracked the fish head nor extended its head to investigate it, however it did have a dazzle reflex, and the rest of the NEx was normal. Based on the NEx, the neuroanatomical localization was the central visual system (somewhere between the eye and visual cortex) bilaterally. Severe bilateral hydrocephalus was identified on MRI examination performed after 25 days in care, and she was ultimately deemed non-releasable.

#### 3.4.2 Year 2

Three animals (M, T, and G) appeared weak, with some decreased motor responses on repeated exams. Animal M required stronger than usual tactile stimulation with forceps to elicit a conscious response and withdrawal of the limb; on repeated examination, it also had reduced motor responses (on the handstand, wheelbarrow, pelvic limb tone, and thoracic flipper digital flexion tests). Animal G seemed dull, weak with short strides; and had minimal response bilaterally on visual field testing. Animal T repeatedly had reduced response to cutaneous stimulation and wheelbarrowing, and it had decreased grasp and pelvic limb tone on one NEx. All three animals were diagnosed with primary malnutrition and eventually considered healthy enough for release. The deficits identified on examination were considered secondary to systemic illness.

## 4 Discussion

In this study we developed a hands-on NEx for phocid pups, based on the standard veterinary protocol used in domestic species. The examination takes 9–12 min to complete depending on the temperament of the animal. We were able to use the standard veterinary protocol as a basis for the NEx, but anatomical differences between HS pups and domestic species necessitated development of novel test methods, particularly for assessing gait and posture, but also excluded tests like pupillary light reflex. Novel test methods included the sloping ramp, banana pose, handstand and manus grasp. The dangling fish head proved useful for evaluating mentation, vision and vestibular system, by observing the face, head and neck movement. As the animals were free-ranging, behavioral responses to stimulation limited use of some hands-on tests, such as facial reflexes elicited by tactile stimulation, and the menace response. Repeating neurological function tests within the same animal, and across many animals, enabled assessment of test consistency and reliability. Bases on these data, a phocid NEx protocol was developed. The protocol was applied to three neurologically compromised animals, for which a neuroanatomical localization was determined; this localization was confirmed through MRI and/or necropsy.

Differences in neural function, and hence the NEx, reflect species-specific anatomy and function. Phocids have distinctive aspects of gait and posture that are specialized for the marine environment but retain aspects for some terrestrial functions. Thus, gait and posture are best assessed both in and out of water. Observation in the water is possible to some extent from above the water surface but could be optimized by using an underwater viewing window or camera. Key anatomical adaptations in phocids include thoracic limbs that are shortened proximally and have elongated phalanges that project laterally as flippers (21). On land, the thoracic limbs move mainly in the cranio-caudal direction and are used for grasping the terrain during trunk protraction, whereas in the water they have a strong adduction-abduction component for steering. The pelvic limbs are also shortened, particularly the femur, which is directed laterally while the distal limb is directed caudally. There is limited movement of the coxofemoral joint, and the seal propels itself by oscillating the caudal body, including the pelvic limbs, horizontally (laterally) (21–23). All limbs can move independently.

The HS pups evaluated in this study were free-ranging and thus unaccustomed to, and potentially fearful of or stressed by, humans approaching and handling them, making hands-on assessment inherently difficult. This was especially so for cranial nerve assessment as handling around the head could be particularly threatening. Although some pups were relatively tractable, others were less amenable. It is worth noting that in free-ranging animals unaccustomed to being handled, tractability may signify a neurological deficiency worthy of investigation. Animal head movement and vocalization unrelated to the examination limited our ability to assess motor responses to facial tactile stimulation, menace response, and dazzle reflex. Such tests can and should still be attempted on such animals, and a positive response may yield useful information (high specificity), but a negative outcome does not necessarily indicate loss of function (12).

In neonatal animals, the maturity of the nervous system must also be considered when assessing their ability to respond to stimuli. Phocids, such as harbor seals, are precocial animals, born with the ability to move and function well at birth including swimming (24) and, as such, age is likely less of a factor in the NEx.

### 4.1 Observation and hands-on neurological examination

Observation of functions (such as mentation, CNN, posture and movement) in animals unaccustomed to being handled is a key aspect in the neurological examination of HS pups (14). We particularly sought to enhance the neurological assessment of HS pups by adding key hands-on techniques based on those used in the NEx protocol in domestic species. In our study, all tests assessed some aspect(s) of sensory input (e.g., vision, touch, proprioception, nociception) and motor output (e.g., movement of one or more body parts). Not all neurological tests were completed in all animals. Reasons included the following: (a) animal temperament—some animals were non-compliant, wary, or fractious. (b) some neurological tests were tried but abandoned because the findings were too difficult to discern (e.g., pupillary light reflex (PLR), menace response) or were unreliable (e.g., hopping); (c) some neural responses, such as gaze fixation, were identified as potentially useful only partway through testing; hence, only some animals were assessed for those responses.

Assessing symmetry is a key aspect of the NEx. Many tests evaluate and compare bilateral morphology and function (e.g., thoracic limb responses, CNN responses). A repeatable unilateral decrease or absence (often on multiple tests) is considered to represent asymmetric decreased neurological function. If a peripheral cause is excluded, asymmetry can correlate with an ipsilateral spinal cord or caudal brainstem lesion, or a contralateral forebrain lesion (1, 17). For example, the lateralizing signs of left visual deficit in animal P correlated with her right-sided forebrain lesion.

Other than the individuals described in the case summaries, the pups’ mentation was appropriate based on observation of their responses to environmental stimuli. It should be noted that decreased responses to stimulation and weakness can occur with either neurological dysfunction or systemic illness (25) as was likely in 3 year 2 pups (M, T, and G). Systemic causes of lethargy include premature birth, malnutrition, developmental issues, and diseases affecting various body systems (25).

#### 4.1.1 Cranial nerves

In HS pups, a full cranial nerve assessment was challenging due to active head movement and vocalizations, with accompanying changes in facial movement. The HS pups were compliant in only 20% of the NEx (having minimal movement or vocalizing). Observing vocalization provides useful information on function of multiple cranial nerves, such as those involved in facial movement (eyelids, vibrissae, maxillary skin all supplied by branches of CN VII), masticatory muscle function (jaw opening and closing—CNN V and VII), laryngeal function (phonation—CNN X and XI), and tongue function (CN XII). However, ongoing vocalization made it challenging to assess menace response (CN II and VII) and response to tactile stimulation (CN V) around the nares and mystacial vibrissae. Visual field testing was reliable and indicated intact visual pathways from the eye to rostral colliculus (midbrain) and stimulation of motor systems (tectospinal tract) to cause neck flexion. Observation of visual tracking permitted assessment of CNN II, III, IV and VI, and observation of globe retraction during blinking aided in assessing CN VI. Facial nerve function was readily assessed by observing facial symmetry and movement (e.g., during vocalization, blinking, and nares movement during respiration). In dogs and cats, to help assess CN IX and X, the gag reflex is elicited by manually stimulating the oropharyngeal area with the fingers and feeling for pharyngeal contraction and tongue retropulsion; we did not attempt this test in HS pups.

The PLR was difficult to assess due to the dark color of the iris and miotic pupil even in dimmed light. As diving animals, HS have irises that are stenopeic (extremely miotic) (26). A longer dark adaptation time (>5 min) before testing the PLR may lead to more obvious mydriasis and hence obvious positive results. Conversely, overt mydriasis would be expected in an animal that has a significant lesion of the eye, CN II, post-chiasmatic visual pathway, midbrain, or parasympathetic portion of CN III; complete mydriasis has been noted in blind pinnipeds (personal observations CF). It may have been present in animal P but was not recorded. When investigating the suspended fish head, presumably the pups are using vision, olfaction and tactile sensation through the mystacial vibrissae (27). However, this test may be less conclusive in younger animals (< approximately 3–4 weeks of age) who are not yet eating and do not yet identify fish as a food source. In conclusion, much of the assessment of the CNN in HS pups, was best done by observation with minimal hands-on stimulation, particularly in fractious animals.

Vestibular function affects posture and motor function of the whole body and was assessed in a variety of ways, including by observation of head posture and gait. In the hands-on NEx, the righting reflex, banana pose, the suspended phase of the handstand, and head extension to the fish head tests were used. The last two tests elicit strong proprioceptive input from receptors in the inner ear and from the neck originating from the cranial cervical muscles (spinovestibular tracts) (1, 18). In mammals, head position determines eye position, and head movement causes flicking eye movement (physiological nystagmus or vestibulo-ocular reflex). In dogs, physiological nystagmus can be elicited either by holding and moving the head laterally or moving the whole animal horizontally (1, 17). Free-ranging HS pups usually would not permit having their heads held; however, by moving the pup horizontally from side-to-side, in some animals, it was possible to see globe movement by its distortion of the upper eyelid. In the last 2 days of testing in year 2, it was noted that during horizontal rotation of the pup the eyes would fixate on a point, thus moving in the direction opposite to head movement. This was followed by a quick head and eye movement in the direction of rotation, and repeated gaze fixation on another visual target. This type of saccade would utilize input via CNN II (vision), VIII (head proprioception), cranial cervical muscle spindles, and output to CNN III, IV, VI and neck muscles. The consistency of this function requires further evaluation.

#### 4.1.2 Posture and gait

Normal posture and gait involve a variety of neural functions, including the proprioceptive sensory input system, cerebellum and vestibular systems, and the motor systems (1, 17). We assessed these co-dependent functions both by observation of the animal’s posture and spontaneous movement and putting the animal into different positions to assess their strength, coordination, and balance in the head, neck, trunk, and limbs.

Phocid locomotion on land is mainly by vertical undulations of the trunk that propel the whole body forward, known as galumphing, often with simultaneous use of the thoracic limbs protracting, planting, and retracting to aid forward propulsion. As this mode of locomotion differs greatly from that in non-phocid pinnipeds, we both adapted and developed novel methods of neurological assessment.

Proprioception was assessed using the handstand, righting reflex, and banana pose and observing whether the HS pups sensed slippage on the sloping ramp and grasped the surface by flexing the thoracic limb digits. The sloping ramp causes gentle, slow sliding, which stimulates proprioceptors (especially muscle stretch receptors in the thoracic limb girdle) causing 85% of pups to flex their digits and grip with their claws. This procedure is somewhat analogous to the reflex step test in dogs and cats, in which a piece of paper is placed under each foot and gently pulled laterally. Doing so stretches extrinsic muscles of the proximal limb (especially adductor muscles), stimulating sensory nerves of the PNS, which then stimulates local spinal reflex arcs as well as projecting to the cerebellum. The cerebellum coordinates the function of brainstem motor nuclei to induce stepping, which brings the limb back under the center of gravity. In HS pups, the sloping ramp test stimulates motor output comprising digital flexion and gripping and may also activate stepping movements.

Based on the canine NEx, we assessed hopping, knuckling and tactile placing in the HS pups. In dogs, hopping in each limb, is achieved by moving them laterally. The elongated phalanges of phocids project laterally, and so we moved them in a more cranial direction, similar to limb movement during terrestrial locomotion. However, less than half of the animals tested exhibited any response, thus hopping was not found to be reliable. “Knuckling” (turning the foot over so the animal stands on the dorsum) was also not a useful test. The HS pups were often not tolerant of their flippers being handled and tried to bite the handler. Tactile placing, where the animal is held up and moved so that the dorsum of the foot touches against a tabletop, was also not useful. In dogs, this stimulates lifting and placing the foot on the surface. We attempted tactile placing in several animals, but they did not try to place the appendage on top of the table.

Assessing for paresis was done by observation and while handling the HS pups. In the latter, the examiner could determine strength both during spontaneous and stimulated movement in each limb and the tail.

Coordination of posture and movement largely reflects cerebellar function (1, 24). In HS pups, it can be assessed by observation of posture and gait. Dysfunction can result in ataxia, spasticity, tremor and imbalance. Inducing neck extension (e.g., to the fish head and during the handstand) requires cerebellar coordination of neck muscle function (flexors and extensors). Cerebellar dysfunction could result in a head tremor. This is somewhat analogous to an intention tremor in domestic species, in which tremors may increase when the animals intend a motor activity (1, 17). Signs of cerebellar dysfunction can also be identified with tests that challenge vestibular input and require coordination of unusual movements, such as righting reflex and banana pose.

#### 4.1.3 Cutaneous sensation and nociception

Sensory input from the body surface receptors was best assessed using rounded sponge forceps to lightly squeeze the flippers and tail when the animal was not looking at the assessor and observing the animal’s responses. Motor responses to this stimulus ranged from twitching to actual withdrawal of the appendage. Stimulation of the one pelvic limb flipper could also lead to withdrawal of both pelvic limbs. In terrestrial mammals, noxious stimulation such as pinching the foot with forceps elicits a withdrawal reflex. This reflex is present even if there is a complete disruption to the spinal cord cranial to the spinal cord origin of the limb nerves (the spinal cord intumescence). However, an animal with such severe spinal cord damage will have no conscious response to noxious stimulation, as the nociceptive signal is blocked from reaching the brain. The HS pups tested in this study had intact spinal cords, so only a light, pinch stimulus was required to produce a reflex withdrawal. Some animals also turned to look at the stimulus, indicating a conscious response.

#### 4.1.4 Spinal reflexes

Spinal reflexes use local neural reflex arcs and do not require input from higher centers. They are present at birth (“hard-wired”) and need not be learned. In domestic species, commonly tested spinal reflexes include patellar, withdrawal, cutaneous trunci (skin twitch), and perineal reflexes. Reflex testing in HS pups was more challenging because of their relatively short limbs, unique anatomy and temperament. However, the withdrawal reflex was elicited (see section 4.1.2) in response to squeezing the flippers and tail in 88% of animals. In 92% of animals, thoracic flipper digital flexion (manus grasp) was elicited by stroking the manus. Whether this is a true spinal reflex is unclear, but it is found to occur in neonates and thus can likely be considered hard-wired and therefore, a reflex.

### 4.2 Consistency and reliability of neurological function tests

In year 2, pups were examined twice to determine (a) whether the results of individual neurological function tests were repeatable and hence consistent within individual animals (Table 3) and (b) whether a specific test was reliable producing consistent results in many animals (Table 4). High consistency within an animal, and reliability of individual test results across many animals, validated inclusion of that test in the neurological examination protocol.

### 4.3 Recommended NEx protocol for harbor seal pups

The NEx protocol for pups is summarized in Figure 5 (NEx Protocol data record sheet). Any neurological assessment protocol should evaluate all regions of the nervous system, both peripheral and central. Table 5 lists the main divisions of the nervous system and tests that can assess function in those regions.

**Table 5:** Neural functions associated with major regions of the nervous system.

Region of the nervous system	Associated neural function
Forebrain	Mentation, behavior, proprioception, skilled motor function—e.g., use of whiskers, CNN I and II, processing of sensory stimuli (tactile, nociception, vestibular, hearing, vision)
Brainstem	Mentation, proprioception, motor function for gait and posture, CNN III-XII
Cerebellum	Proprioception, coordination of motor function, regulation of movement and tone, balance (vestibular function)
Spinal cord	Sensory input: including proprioception, nociception. Motor function: UMN tracts for voluntary movement, gait and posture, origin of LMN, spinal reflexes
Peripheral nervous system	Spinal and cranial nerves and their reflexes, sensory (proprioception, tactile, nociception), muscle tone

This information can aid in using findings of the neurological examination to identify the region of the nervous system compromised by disease. For example, finding that an animal is dull/obtunded, has proprioceptive deficits or paresis, and has various cranial nerve deficits could indicate that the animal has a brainstem lesion. CNN, cranial nerves; UMN, upper motor neurons; LMN, lower motor neurons. UMN = motor tracts arising in the brain, and connecting via the brainstem or spinal cord, to LMN. LMN arise in the brainstem or spinal cord and connect via the cranial or spinal nerves to muscles.

Although we did not actively assess the function of the autonomic nervous system, it could be tested by assessing pupil function, particularly if mydriasis is present (CN III lesion), tear and saliva production (CNN VII, IX), heart and respiratory rate, gut function, and elimination (urination and defecation). In dogs, the cardiorespiratory, gut and elimination functions are innervated by CN X and thoracic, lumbar, and sacral spinal nerves (1, 17, 28).

### 4.4 Case studies

In year 1, three animals (L, P, and N) were noted to have neurological abnormalities and used as case studies. Although the full NEx protocol was not established in year 1, the visual field test, handstand, and righting reflexes were seminal in identifying neurological deficits and developing a neuroanatomical localization. Both animals P and N had extensive forebrain lesions yet only mild neurological deficits, mainly of vision. Similarly, in domestic species, relatively minimal neurological deficits may be seen with large unilateral forebrain lesions, especially if the lesion occupies the lateral or caudal cerebrum. This observation is consistent with the fact that major motor centers for gait and posture are located in the brainstem in domestic veterinary species (1, 6, 17, 28, 29). In a previously published report from 2005, a 5-month-old stranded HS had no observed neurological deficits even though most of the right cerebrum was absent, and the left cerebellum was hypoplastic (30). In that animal, a hands-on NEx may have revealed visual and possibly nociceptive deficits, as well as coordination deficits. In our study, Animal L had overt cerebellar signs yet only minimal changes on MRI, which suggested inflammatory, infectious, or toxicologic CNS disease. He improved following antimicrobial treatment which suggests an infectious etiology. In year 2, three animals (M, T, and G) were identified as having weakness, reduced mentation, or both. However, these signs are not specific for neurological disease and can also result from systemic illness or malnourishment, which can occur with premature separation from their mothers.

The aim of any neurological examination is to identify the regions of the nervous system that are functioning normally and those that are compromised by disease. Both normal function and dysfunction are used in lesion localization (Figure 5, page 3). In domestic species, neuroanatomical localization is used in conjunction with signalment (age, breed, sex) and history to develop a list of the most likely causes of the neurological problem (differential diagnosis). However, in free-ranging animals, although history is limited, data collected at the site where the animal was found and at the rehabilitation facility is invaluable. Lesion localization is essential for building a list of differential diagnoses and deciding which diagnostic tests (e.g., serology, toxin levels or imaging) are most appropriate. To help with lesion localization, Table 5 briefly summarizes the regions of the nervous system and tests for assessing their functions.

Neurological deficits in HS pups were also noted in the study by Lian et al. (14). In free-ranging-born HS pups undergoing rehabilitation at TMMC, HS pups with significant *in utero* exposure to mercury had decreased movement and response to cutaneous stimulation. The pinnipeds in the San Francisco Bay area are particularly susceptible to mercury intoxication caused by decades of mining activity contaminating the Bay Area with mercury and other chemicals (31), however numerous other etiologies are also possible including other neurotoxic biotoxins (14, 32–35). Other neurologic diseases previously reported in harbor seal pups include infectious diseases such as influenza (36), morbillivirus (37), herpesvirus (15), parvovirus (38), protozoan parasites (39) and mixed bacterial infections (40), thiamine deficiency (41), developmental anomalies (42, 43), including atlantoaxial subluxation (44), Dandy-Walker-like malformation (45) and trauma (46).

### 4.5 Limitations of study

The primary limitations of this study included: multiple examiners performing the NEx, limitations imposed by video technique, and animal temperament.

Three assessors worked together while learning the testing techniques; however, reviewing the videos revealed differences in testing technique. Video limitations primarily occurred if a video was taken at the wrong angle, so the animal’s response to a test was not visible. Thus, while a response might have been obvious during testing, it may have appeared inconclusive when subsequently assessing videos. Additionally, assessing the outcome of the NEx by reviewing videos may be impractical in a busy clinical setting. Performing multiple NEx facilitates proficiency with the techniques and helps the examiner become familiar with the range of normal responses. With practice, the clinician will be able to do a hands-on neurological assessment in HS pups in about 10 min, especially when already familiar with this species and age group.

Animal temperament (movement and lack of compliance preventing visualization of a clear response) was the main reason that tests were classified as inconclusive. This factor primarily affected evaluation of cranial nerve function, much of which was assessed by observing the animal’s response to a specific stimulus such as touch or vision. Ongoing head movement and vocalization caused spontaneous movement of eyelids and nares and the jaw.

In year 1, three animals (L, P, and N) were found to have neurological deficits and used as case studies for testing the NEx protocol. The full protocol had not been developed at that stage, representing a limitation on interpreting the results. However, key tests that were seminal in evaluating these animals were utilized (as noted in section 4.4) and the subsequent neuroanatomical localization was confirmed.

### 4.6 Future directions

In the future, using the NEx protocol in both neurologically normal and neurologically compromised animals as well as in other phocids and marine mammals would permit further optimization and expansion of the range of tests.

In neurologically normal HS pups, ongoing development of the NEx protocol could permit assessment of new tests such as gaze fixation, and protocol modifications may improve the usefulness of tests such as hopping and pelvic flipper fan. Underwater assessment of swimming may help identify deficits in posture and motor function, or potentially CNN deficits. For example, did Animal P, who was blind in the left eye due to porencephaly, swim with her eyes closed underwater?

Further validation of this NEx could be achieved by using it at other facilities. Only one geographical site (TMMC) was used here, but the pups examined in this study had been stranded over a 900-kilometer range of Central and Northern California, representing a variety of causes and stranding locations. The techniques described here should be applicable to habituated pups under professional care. As those animals may be more accustomed to handling by people, certain tests, such as CNN evaluation, may yield more consistent results.

It should be noted that older HS and other pinnipeds can be dangerous and impose risks for human handlers. The HS in this study were pups weighing less than 30 kg and with varying degrees of aggression in response to handling. The observational aspect of the NEx will be of particular use in assessing larger phocids and pinnipeds.

The NEx protocol should (a) help identify animals that have neurological dysfunction and (b) localize the lesion in the nervous system, so that appropriate diagnostic testing and case management can be undertaken. Its efficacy would be validated by assessing pups with a variety of neurological deficits to determine the neuroanatomical localization, and then confirmation of lesion localization either by MRI or necropsy. The protocol could also be extrapolated to other smaller phocids and pinnipeds affected by trauma, infectious/inflammatory agents, developmental anomalies or other causes. It will help identify those animals with mercury-induced developmental deficits and animals affected by other neurotoxic biotoxins, such as domoic acid (14, 32–35).

## 5 Conclusion

Lian et al. (14) described an observational protocol for assessing neurological function in HS pups. We have now developed and evaluated a standardized, hands-onneurological examination protocol for HS pups. Aspects of this protocol may be applicable to other marine mammals. The protocol combines observation and hands-on testing, as is important in free-ranging animals in which handling options are often limited. Future directions should include testing more animals with neurological dysfunction and correlating those findings with lesion localization and disease agent.

## Data Availability

The original contributions presented in the study are included in the article/supplementary material, further inquiries can be directed to the corresponding author.
